# The Spatiotemporal Regulation of cAMP Signaling in Blood Platelets—Old Friends and New Players

**DOI:** 10.3389/fphar.2015.00266

**Published:** 2015-11-10

**Authors:** Zaher Raslan, Ahmed Aburima, Khalid M. Naseem

**Affiliations:** Centre for Cardiovascular and Metabolic Research, Hull-York Medical School, University of Hull, Hull, UK

**Keywords:** platelets, adenylyl cyclase, cAMP, A-kinase anchoring proteins, protein kinase A

## Abstract

Atherothrombosis, the pathology underlying numerous cardiovascular diseases, is a major cause of death globally. Hyperactive blood platelets play a key role in the atherothrombotic process through the release of inflammatory mediators and formation of thrombi. In healthy blood vessels, excessive platelet activation is restricted by endothelial-derived prostacyclin (PGI_2_) through cyclic adenosine-5′-monophosphate (cAMP) and protein kinase A (PKA)-dependent mechanisms. Elevation in intracellular cAMP is associated with the control of a number of distinct platelet functions including actin polymerisation, granule secretion, calcium mobilization and integrin activation. Unfortunately, in atherosclerotic disease the protective effects of cAMP are compromised, which may contribute to pathological thrombosis. The cAMP signaling network in platelets is highly complex with the presence of multiple isoforms of adenylyl cyclase (AC), PKA, and phosphodiesterases (PDEs). However, a precise understanding of the relationship between specific AC, PKA, and PDE isoforms, and how individual signaling substrates are targeted to control distinct platelet functions is still lacking. In other cells types, compartmentalisation of cAMP signaling has emerged as a key mechanism to allow precise control of specific cell functions. A-kinase anchoring proteins (AKAPs) play an important role in this spatiotemporal regulation of cAMP signaling networks. Evidence of AKAP-mediated compartmentalisation of cAMP signaling in blood platelets has begun to emerge and is providing new insights into the regulation of platelet function. Dissecting the mechanisms that allow cAMP to control excessive platelet activity without preventing effective haemostasis may unleash the possibility of therapeutic targeting of the pathway to control unwanted platelet activity.

## Introduction

Blood platelets play a key role in haemostasis through binding to sites of vascular injury to form a primary haemostatic plug. However, uncontrolled platelet activation is intimately linked to thrombotic events associated with the rupture of atherosclerotic plaques. Controlling inappropriate platelet activation is critical to protecting against thrombotic episodes. Primary inhibitory mechanisms are mediated by endothelial derived-nitric oxide (NO) and prostacyclin (PGI_2_), which stimulate soluble guanylyl cyclase (sGC) and adenylyl cyclase (AC) leading to activation of cyclic guanosine monophosphate (cGMP) and cyclic adenosine monophosphate (cAMP)-mediated signaling pathways, respectively (Smolenski, 2012). cAMP/cGMP-dependent protein kinases phosphorylate target proteins that are associated with controlling platelet activation while multiple phosphodiesterases (PDEs) hydrolyse cyclic nucleotides to terminate signaling. The cAMP and cGMP signaling pathways have overlapping target specificity, but also synergise indicating diversity in target selection. In many cell types the enzymes that generate, propagate and terminate cAMP signaling are organized into restricted microdomains that focus the activity of the cAMP signaling cascade to specific substrates (Tasken and Aandahl, 2004; Scott and Pawson, 2009; Stangherlin and Zaccolo, 2012). This compartmentalisation of cAMP signaling allows stimulus-specific control of distinct cell functions. Given the central importance of cAMP signaling to the control of platelet function, surprisingly little is known about the molecular mechanisms that coordinate the synthesis and hydrolysis of cAMP, the precise molecular targets of cAMP signaling and how these targets contribute to the control of specific platelet functions. In this perspective article, we provide an overview of cAMP signaling and examine emerging concepts of compartmentalization in platelets.

## The cAMP Signaling System in Platelets

**FIGURE 1:**
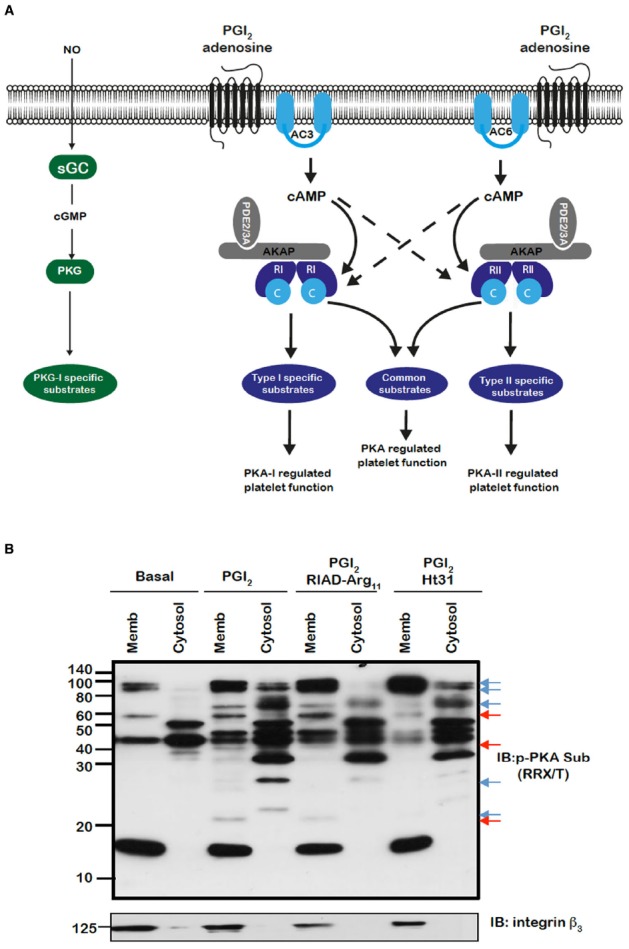
**(A)** Model for the control of platelet function by cAMP signaling in platelets. Segregated pools of cAMP are formed by the individual isoforms of AC. The cAMP generated may target individual PKA isoforms. AKAPs act to focus isoform specific PKA activity to individual substrates that are linked to distinct platelet functions. The AKAPs may also act to localize PDE isoforms in manner that controls specific signaling events. **(B)** PKA substrates are differentially distributed and targeted by PKA in an AKAP-dependent manner. Washed platelets (5 × 10^8^/mL) were left untreated or pre-treated for 60 min with RIAD-Arg_11_ (2 μM) or stHt31 (2 μM), which target PKA-I and PKA-II specific AKAPs respectively, followed by PGI_2_ (50 nM) for 1 min. Platelets were lysed then subjected into subcellular fractionation (20,000 × *g*, 90 min at 4°C) to separate platelet membranes. Membrane and cytosol fractions (20 μg) were analyzed by SDS-PAGE and immunoblotting using phosphoPKA substrate antibody and integrin β as a membrane marker. Blots are representative of three independent experiments. Membranes were visualized using enhanced chemiluminescence. Red and blue arrows represent AKAP sensitive bands in membrane and cytosolic bands respectively.

The binding of PGI_2_, prostaglandin E_1_ and adenosine to their receptors on platelets leads to the elevation of intracellular cAMP through the activation of AC. While there are nine membrane bound isoforms of AC, human platelets express only AC3 and AC5/6 (Rowley et al., 2011; Burkhart et al., 2012; Cooper and Tabbasum, 2014). The roles of these individual AC isoforms and whether they are linked to specific receptors or stimuli are unknown. Increased cAMP leads to the activation of protein kinase A (PKA), the foremost effector of cAMP signaling in platelets. The holoenzyme of PKA is an inactive heterotetramer composed of two regulatory (R) and two catalytic (C) subunits. Platelets express all the known isoforms of the regulatory and catalytic subunits (RIα, RIβ, RIIα, RIIβ, Cα, Cβ, and Cγ) (Pidoux and Taskén, 2010; Burkhart et al., 2012), indicating that platelets likely possess multiple variations of the two PKA subtypes, PKA-I and PKA-II, which have distinct biochemical properties. The binding of four cAMP molecules to their binding sites on PKA, result in a conformational change unleashing the catalytic subunit and consequently the phosphorylation of adjacent protein substrates. To date sixteen substrates have been characterized in platelets, although a recent proteomic study suggests that over 100 substrates may be present (Beck et al., 2014). These substrates can be categorized into proteins involved in regulating: (i) shape change (actin polymerization), (ii) calcium signaling, or (iii) integrin activation (Schwarz et al., 2001; Raslan and Naseem, 2014). The phosphorylation of these proteins by PKA is proposed to be the primary mechanism by which prostacyclin modulate platelet activation. As platelet pass through the circulation they are marginalized to the periphery of the vessel facilitating constant exposure to endothelial-derived PGI_2_. Therefore it is likely that the default status of platelets in the circulation is one of elevated cAMP and activated PKA, which maintains the cells in a quiescent state. However, to facilitate platelet activation at sites of vascular injury the inhibitory effects of basal cAMP signaling must be overcome. Upon activation, platelets generate and release soluble factors that act in a paracrine fashion to promote platelet activation through the inhibition of cAMP signaling. Platelet-derived adenosine diphosphate (ADP) inhibits AC activity through binding to Gαi-coupled P2Y_12_ that blocks cAMP synthesis. Simultaneously, thrombin and thrombospondin-1 activate PDE3A, leading to reduced intracellular cAMP (Gachet et al., 2006; Zhang and Colman, 2007; Roberts et al., 2010). Together, these mechanisms act to reduce the threshold for platelet activation and ensure rapid recruitment of platelets to vascular lesions. An imbalance in this dynamic system can have profound effects on platelets function as exemplified by several recently identified congenital disorders that affect cAMP production (Geet et al., 2009). A Gαs hypofunction mutation is associated with platelet hyperactivity (Freson et al., 2009). Conversely, a Gαs gain-of-function mutation is reported to lead to a trauma-related bleeding tendency (Freson et al., 2003). Consistent with these observations, a recently identified mutation in the gene encoding the catalytic subunit of PKA leads to macrothrombocytopenia with the homozygous patients showing a bleeding tendency (Manchev et al., 2014). The control of cAMP signaling and therefore the phosphorylation of key PKA substrates is mediated by both protein phosphatases and several PDEs through the hydrolysis of cAMP into 5′-AMP. Platelets express two cAMP hydrolysing enzymes, PDE2A and PDE3A (Dickinson et al., 1997; Schwarz et al., 2001; Rondina and Weyrich, 2012). However, the phosphatases that dephosphorylate PKA substrates are unknown. Thus, platelets possess a complex cAMP signaling network that includes multiple isoforms of AC, PKA and PDE (Figure 1A), but molecular control and integration of this network is required to maintain the balance between haemostasis and thrombosis.

## Spatiotemporal Regulation of cAMP Signaling—the Role of AKAPs

**FIGURE 2:**
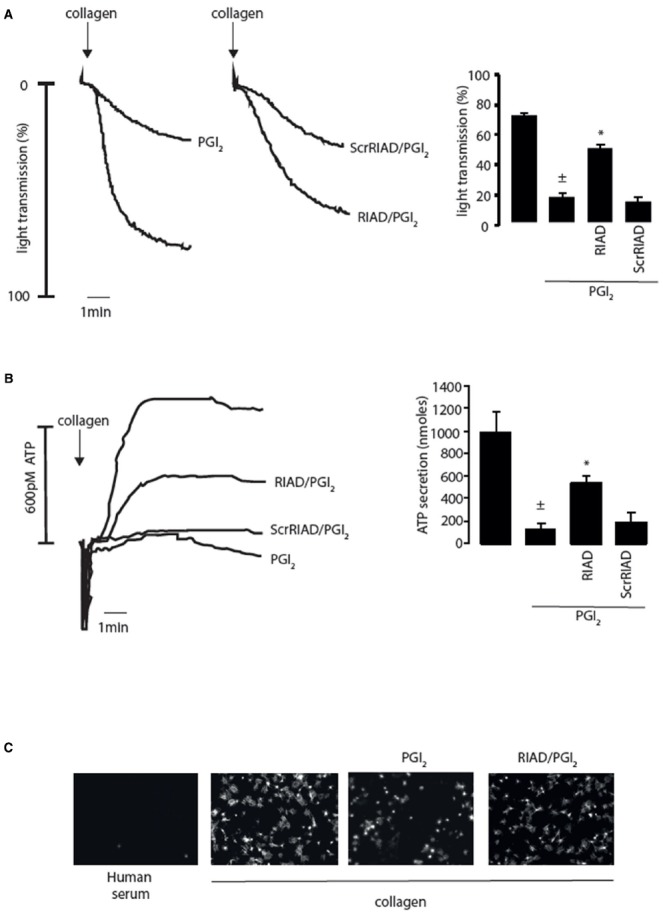
**Inhibition of platelet activity by prostacyclin (PGI_2_) requires PKA-I/AKAP interactions. (A)** Washed platelets (2.5 × 10^8^/ml) were left untreated or pre-incubated with RIAD-Arg_11_ (2 μM) or scrRIAD-Arg_11_ (2 μM) for 1 h followed by addition of PGI_2_ (50 nM) for 1 mine and then stimulated with collagen (5 μg/ml) for 4 min under stirring conditions. (Ai) representative aggregation traces. (Aii) Collated data of four independent experiments expressed as mean ± SEM. ± *p* < 0.01 compared to absence of PGI_2_; *p < 0.05 compared to PGI_2_ alone. **(B)** As in (A) except platelet ATP secretion was measured by the signal released by a Luciferin-Luciferase reaction using a Chrono-log Lumi-aggregometer. (Bi) representative ATP secretion traces. (Bii) Collated data of four independent experiments expressed as mean ± SEM. ±*p* < 0.01 compared to absence of PGI_2_; *p < 0.05 compared to PGI_2_ alone. **(C)** Washed platelets (5 × 10^7^/ml) were placed on human serum (i) or collagen-coated coverslips (50 μg/ml) (ii–iv) for 60 min at 37°C in the absence (ii) or presence (ii) PGI_2_ (100 nM). In (iv) washed platelets were first pre-treated with RIAD-Arg_11_ (2 μM) for 1 h followed by addition of PGI_2_ (100 nM). At the end of the incubation period, non-adherent platelets were removed by washing with PBS. The remaining adherent platelets were fixed, permeabilized with 0.1% Triton, and stained for F-action with TRITC-conjugated phalloidin then visualized with fluorescence microscope. Scale bar is 5 μm.

The spatiotemporal control of cAMP signaling is influenced by the opposing functions of localized AC and PDE isozymes and the scaffolding properties of A-kinase anchoring proteins (AKAPs). The cAMP signal is conveyed through assembly of macromolecules that constrain cAMP signaling to specific regions in the cell. The formation of multiple cAMP signaling hubs where individual isoforms of PKA interact with anchoring proteins that focus PKA activity on specific substrates, allows the control of specific biological functions in response to distinct stimuli (Tasken and Aandahl, 2004; Scott and Pawson, 2009; Stangherlin and Zaccolo, 2012). Early studies reported that PKA substrates were specifically localized in distinct subcellular compartments of blood platelets (El-Daher et al., 1997, 2000). Our own studies support these observations with distinct phospho-PKA substrates found in cytosolic and membrane fractions of platelets (Figure 1B). Consistent with the detection of PKA substrates in multiple cell compartments we found using both subcellular fractionation and immunostaining experiments that PKA-I and PKA-II are differentially localized in platelets. PKA-RI was found primarily at the cell periphery, while PKA-RII was clustered within the cytosol (Raslan et al., 2015). We found that membrane lipid rafts allow for further compartmentalization of cAMP signaling in platelets. For example, a pool of AC isoform 5/6 and PKA-I, but not PKA-II, are localized to these membrane micro-domains in platelets where the rafts act to constrain PKA activity (Raslan and Naseem, 2015). Thus, PKA signaling in platelets, like T-cells, dendritic cells and cardiomyocytes is compartmentalized to membrane fractions in close proximity to the site of cAMP generation (Ruppelt et al., 2007; Delint-Ramirez et al., 2011; Schillace et al., 2011; Burgers et al., 2012). The question then arises as to how these cAMP signaling compartments are organized and configured in platelets. An elegant study using a chemical proteomic approach indicated the presence of localized and active cAMP signaling nodes, which were dynamic in nature with PKA activity changing within distinct signaling complexes in response to platelet activation.The formation of these complexes was attributed to the presence of several AKAPs, although the precise composition of these complexes or how they were convened was not extrapolated (Margarucci et al., 2011). AKAPs are structurally diverse scaffolding proteins that interact with PKA and target it to a specific subcellular compartment through a unique targeting domain. All AKAPs possess a highly conserved PKA binding domain. This is an amphipathic helix, which can bind to the docking and dimerization domain (D/D domain) of the PKA regulatory homodimer (Carr et al., 1991; Kinderman et al., 2006; Sarma et al., 2010). While AKAPs specifically bind PKA, they can act as hubs that coalesce other signaling components including kinases, phosphatases, PDEs, ACs, and even receptors. The assembly of such macromolecules act as signal switches that are intricately regulated spatially and temporally by signal initiators and terminators. Over fifty AKAPs have now been identified and of these fifteen are potentially expressed in platelets (Margarucci et al., 2011; Rowley et al., 2011; Burkhart et al., 2012). Given the potential complexity within the cAMP signaling system, it is likely that AKAPs play a key role in the control of platelet function by cAMP (Figure 1A). Unfortunately, the study of AKAPs in platelets has been limited by an inability to apply standard molecular biology approaches used to characterize AKAPs in many other cells. However, he development of cell permeable disruptor peptides that mimic the sequence of the amphipathic helix of the PKA binding domain and target PKA-AKAP interactions in an isoform-specific manner has allowed us to begin examine this hypothesis (Carlson et al., 2006; Gold et al., 2006; Zhang et al., 2014). These peptides can displace PKA from multi-protein complexes formed by AKAPs allowing the functional relevance of these AKAPs to be evaluated. Focusing on PKA-I we used RI-anchoring disruptor peptide or RIAD-Arg_11_ to demonstrate that a number of cAMP signaling events in platelets were dependent on PKA-I-AKAP interactions (Carlson et al., 2006).Examining PKA signaling events using immunoblotting following subcellular fractionation, we were able to observe a decrease in the intensity of PKA phosphorylation events after stimulating with PGI_2_ in the presence of RIAD-Arg_11_ in both membrane and cytosolic fractions when compared with PGI_2_ alone suggesting that PGI_2_ trigger PKA-I-specific signaling events that are AKAP-dependent in platelets (Figure 1B). This was followed by a number of specialized assays, including aggregation, secretion and spreading to evaluate the role of PKA-I-AKAPs interactions in the inhibitory effect of prostacyclin on platelet function (Figure 2). As expected collagen-induced platelet aggregation was inhibited by PGI_2_, but the inhibitory action was diminished significantly by the presence of RIAD-Arg_11_ (Figure 2A). The secretion of dense granules is a major factor in potentiating platelet aggregation in response to collagen.Luminescence assays demonstrated that PGI_2_ abolished the secretion of ATP from these granules in response to collagen, but again the presence of RIAD-Arg_11_ reversed its inhibitory effects (Figure 2B). Finally platelets were adhered to immobilized collagen to induce a physiological spreading response. In response to collagen, platelets extended filopodia and lamellipodium consistent with spreading (Figure 2C). The presence of PGI_2_ prevented the spreading response without influencing the ability of platelets to adhere. However, pre-treating platelets with RIAD-Arg_11_, allowed platelets to adhere and spread on collagen. Interestingly, in all assays, RIAD-Arg_11_ failed to cause a full recovery from the inhibitory effects, with the remaining effects of cAMP potentially caused by PKA-II, which would be insensitive to the effects of RIAD. While the data produced using the peptides should be interpreted with caution, our observations suggest that PKA-I-AKAP interactions are required for optimal PGI_2_ inhibition of collagen-induced platelet activation and that PKA-I and PKA-II have non-redundant roles in human platelets.

The signaling events driven by compartmentalisation of PKA isoforms are finessed by the PDEs (Rondina and Weyrich, 2012). Using FRET, Lissandron et al. (2006) demonstrated that distinct pools of cAMP could be shaped by specifically localized PDE isoforms (Lissandron et al., 2006; Stangherlin and Zaccolo, 2012). While the subcellular localisation of platelet PDEs is not clear there is evidence for potential non-redundant roles in controlling cAMP signaling. Pharmacological inhibition of PDE2A resulted in increased basal cAMP, while inhibition of PDE3A blocked platelet aggregation, reduced Ca^2+^ mobilization and increased vasodilator stimulated phosphoprotein (VASP) phosphorylation (Feijge et al., 2004). These data suggest PDE2A and PDE3A play non-redundant roles, whereby the former controls elevated cAMP levels and the latter maintains a threshold of cAMP levels in pools that control integrin activation and Ca^2+^ mobilization. Interestingly, PKA has been shown to phosphorylate and activate of PDE3A in platelets (Macphee et al., 1988; Hunter et al., 2009), suggesting a feedback regulatory mechanism within platelets to control cAMP signaling. The molecular composition of this feedback regulatory complex is ill-defined, although preliminary data in our laboratory suggests that phosphorylation of PDE3A is performed by PKA-II in an AKAP-dependent manner (Law and Naseem, unpublished). The localisation of PDE isoforms to specific signaling complexes and PKA substrates is a key area for further characterisation of cAMP signaling in platelets.

## Evidence of Functional AKAPs in Platelets

The concept of isoform-specific PKA substrates in platelets that are regulated by an AKAP is an attractive one, although evidence for their functional roles is still lacking. Platelet AKAPs as reported in proteomic and transcriptome studies include AKAP1, AKAP2, AKAP7(γ), AKAP9, AKAP10, AKAP11, AKAP13, moesin, ezrin, Rab32, BIG2 (brefeldin-A-inhibited guanine-nucleotideexchange protein 2), WAVE-1 (Wiskott–Aldrich syndrome protein verprolin homologous 1), MAP2 (microtubuleassociated protein 2), smAKAP (small-membrane AKAP) and neurobeachin. Of those fifteen AKAPs we were able to verify the presence of several by immunoblotting techniques. To the best of our knowledge only two functional AKAPs have been identified in platelets, small membrane AKAP (smAKAP) and moesin. SmAKAP was identified in platelets and cardiomyocytes by using chemical proteomics. This AKAP specifically targets PKA-I to cellular membrane through myristoylation and palmitoylation anchors (Burgers et al., 2012). Verification of the presence of smAKAP by immunoblotting and its functional role is yet to be determined in platelets. Using a combination of cAMP pull-down, immunoblotting and kinase assays we reported recently that moesin targets PKA-I into platelet lipid rafts where it can phosphorylate its physiological substrate GPIbβ (Raslan et al., 2015). This phosphorylation event contributes to the inhibition of platelet-driven thrombus formation to the adhesive protein von Willebrand factor. This data identifies moesin as the first functionally validated AKAP in platelets, but also indicate GPIbβ as the first PKA-I-specific substrate in platelets. Interestingly, we observed multiple phosphoproteins in platelet lipid rafts that were sensitive to the localisation of PKA-I suggesting that these microdomains may focus PKA-I on several targets required for regulation of platelet function by cAMP. These two studies provide an insight into the potential compartmentalization of cAMP signaling by AKAPs, although more work is required to understand the composition and dynamics of AKAP-regulated macromolecules in platelets.

## Perspective

The cAMP signaling pathway represents the most potent endogenous system for the control of platelet activation through its ability to modulate multiple platelet functions. However, the molecular mechanisms by which cAMP/PKA signaling controls individual platelet functions are unclear. Fundamental to elucidating this issue is an improved understanding of the organization and spatial resolution of the downstream effectors of cAMP in platelets. Emerging data suggests that compartmentalization of key cyclic nucleotide signaling complexes may control aspects of platelet function as evident by the identification of a localized PKG1β-IP_3_R1-IRAG-PDE5 complex that regulate calcium release in response to NO (Wilson et al., 2008). The challenge for platelet biologists is to determine whether this multi-protein complex model can be extended to the cAMP pathway. The advances in both transcriptomics and proteomics have now identified a number of AKAPs that potentially provide spatial resolution and specificity to PKA-mediated phosphorylation events. Our data demonstrating differential localization of PKA-I and PKA-II and compartmentalization of PKA-I by the AKAP moesin to target a specific substrate provide proof of principle that the AKAP hypothesis can be translated to platelets. Interestingly, we have found that the catalytic subunit of PKA is constitutively associated with RhoA (Aburima et al., 2013), PDE3A and IP_3_R1 through AKAP-dependent mechanisms (unpublished), suggesting the presence of numerous individual PKA-AKAP complexes. Unfortunately, platelet research in this area is hampered by an inability to apply conventional gene silencing or overexpression technologies and therefore relies on the identification of native complexes through protein purification. As protein purification techniques become more sensitive, the ability to identify and characterize native complexes increases and may allow functional relevance to be established. Our data with cell permeable disruptor peptides provide one approach to address this issue. However, these peptides are limited in their specificity and only allow resolution of isoform-specific signaling and functional events. Future lines of investigation could involve the identification of individual PKA-AKAP complexes and the application of a new generation of peptide disruptors that discriminate between individual PKA-AKAP complexes (Gold et al., 2013). An important resource for the functional assessment of AKAPs in platelets is genetically modified mice. In the absence of standard molecular biology approaches, mice offer a strategy for examining the role of specific PKA-AKAP complexes on haemostasis and thrombosis *in vivo*. For example, mice heterozygous for Neurobeachin, an established AKAP (Wang et al., 2001), demonstrated altered PKA phosphorylation profile and abnormal platelet dense granules (Nuytens et al., 2013). Detailed examination of platelet sensitivity to cAMP of such mice and their use in adoptive transfer protocols are likely to be key tools for future evaluation of specific PKA-AKAP complexes in the physiological control of platelet function.

The original AKAP hypothesis has now evolved beyond simple anchoring of PKA signaling, with identification of numerous other signaling moieties including phosphatases, PDEs and other kinases coalescing into the complexes (Zaccolo, 2011; Scott et al., 2013). Moreover, these multi-protein signaling hubs are extremely dynamic in nature and have been shown to be both tissue and substrate specific. This particular concept has a specific relevance for platelets where cAMP signaling needs to respond to physiological stimuli that promote platelet activation. Indeed, it may be that the dynamic adjustment of these complexes, in response to agents that promote cAMP generation and platelet agonists, is what determines the threshold for platelet activation and allows effective haemostasis while preventing thrombosis. We believe that the identification of platelet AKAPs through proteomics provides the platform for new lines of investigation in establishing the spatial and temporal organization of cAMP signaling in platelets and how this intersects with platelet activatory signaling.

## Author Contributions

ZR, AA, and KN designed the experimental work, planned and edited the manuscript; ZR and AA performed the experiments; ZR and KN analyzed the data and wrote the manuscript.

### Conflict of Interest Statement

The authors declare that the research was conducted in the absence of any commercial or financial relationships that could be construed as a potential conflict of interest.
